# The Utility of Minimally Invasive Surgery in the Emergency Management of Femoral Hernias: A Systematic Review

**DOI:** 10.3389/jaws.2023.11217

**Published:** 2023-04-21

**Authors:** Paul Shuttleworth, Shariq Sabri, Andrei Mihailescu

**Affiliations:** Department of General Surgery, Tameside General Hospital, Tameside and Glossop Foundation Trust, Ashton-under Lyne, United Kingdom

**Keywords:** emergent groin hernia, emergency, minimal invasive surgery, laparoscopic surgery, femoral hernia

## Abstract

**Background:** Femoral hernias are a relatively rare type of hernia but have a high complication rate, with a high proportion either presenting as an emergency or requiring emergency management. Minimal access surgery has been shown to be safe, with good results, in an elective setting, but there is little published evidence of its utility in an emergency.

**Methods:** A systematic review was conducted searching PubMed, OVID, Embase, and Cochrane reviews for ((Femoral hernia) AND (laparoscop* OR minimal access OR robotic)) AND (strangulat* OR obstruct* OR incarcerat*).

**Results:** 286 manuscripts were identified of which 33 were relevant. 24 were individual case reports, 3 case series, 4 cohort studies or case control series, and 2 high level reviews of National registers.

**Conclusion:** Minimal access surgery can avoid an unnecessary laparotomy for the assessment of hernial contents, especially *via* a TAPP approach. Minimal access repair of femoral hernias as an emergency is feasible and can be done safely with results similar to open surgery but good quality evidence is lacking.

## Introduction

Femoral hernias are a relatively uncommon hernia defined as herniation through the femoral ring, into the femoral canal. They account for around 2%–8% of groin hernias, which is probably an over estimate given that non-operative management is much more common with inguinal hernias, and femoral hernias are over-represented. The femoral ring has relatively rigid borders, and therefore these hernias are prone to strangulation and often present as an emergency, with around 45% operated on as an emergency (1). West et al demonstrated the high complication rate of femoral hernias with 23.2% of patients operated on as an emergency requiring a small bowel resection and their high complication rate is well recognised (2). The relative tightness and rigidity of the femoral ring makes hernia reduction particularly difficult when compared with inguinal hernias. Combined with the high complication rate and likelihood of bowel resection if done as an emergency, the utility of a laparoscopic or robotic approach is questionable. The standard approach to a femoral hernia is either a high pre-peritoneal approach *via* a McEvedy incision, low approach *via* a Lockwood, or trans-inguinal *via* a Lotheissen’s (3). Each have their benefits either allowing easier repair from a low approach, or access to the peritoneal cavity from a high, pre-peritoneal approach. A transabdominal minimal access approach can negate the need for a laparotomy to examine the hernia contents, e.g., after reduction of potentially strangulated small bowel. It can also facilitate management, e.g., resecting ischaemic omentum. The low incidence of femoral hernias makes randomised control trials difficult to perform and the evidence is lacking. The variation in techniques, open, laparoscopic, mesh based or non-meshed based repairs, reflects the lack of high-level evidence for the best operative approach.

Minimal access surgery brings well recognised benefits of less post operative pain, earlier return to function, and in regards to groin hernias may bring lower chronic groin pain with comparable recurrence rates (4,5). A recent meta-analysis of 35 RCTs confirmed these benefits in inguinal hernias, but again failed to demonstrate a benefit in terms of long-term recurrence rates (6). These benefits have been established for inguinal hernias and the current Herniasurg recommendations for elective inguinal hernia repair are that laparoscopic repair should be offered if the surgeon has adequate experience and training (7). Evidence for emergency femoral hernia management *via* a minimal access approach is lacking in comparison with inguinal hernias, and a lot of the recommendations are extrapolated from data for inguinal hernias.

The evidence base supports the use of minimal access surgery (predominantly laparoscopic) in the elective management of femoral hernias (8) and data from the Danish Hernia Database shows a reduction in recurrence of a groin hernia after laparoscopic repair. They demonstrated a high rate of inguinal hernia development after open femoral hernia repair, particularly after McVay procedure.

With the growing availability of cross-sectional imaging and it’s increasing utilisation in an emergency setting (9), we are increasingly more confident of the anatomy and contents of a groin hernia prior to operating, and therefore will be more likely to be aware of the presence of a femoral hernia. The role of laparoscopic repair in the emergent setting needs to be examined.

We performed a systematic review of the evidence base for a minimal access approach when dealing with a femoral hernia presenting as an emergency, to see if it’s use is supported.

## Methods

### Information Sources

A systematic search of OVID, Medline, PubMed, EMBASE, and Cochrane reviews was performed on 4th January 2023. Papers included were read in full and reference sections interrogated to identify any more papers which could potentially have been missed from the search.

### Search Strategy

The following search strategy was used.

((Femoral hernia) AND (laparoscop* OR minimal access OR robotic)) AND (strangulat* OR obstruct* OR incarcerat*).

### Eligibility

#### Inclusion Criteria

Articles were included if the full text was available in English, they related to an adult population (over 16 years old), they involved the management of femoral hernias, *via* a minimal access approach, as an emergency.

After debate, the research group decided to include the 24 case reports found in literature as apart from these, only 4 case series and 5 cohort studies were meeting the eligibility criteria were identified.

#### Exclusion Criteria

Articles relating to elective management. Conference proceedings, reviews. Articles where there was no discrimination between femoral/inguinal hernias, or elective/emergency cases in any of the presented data, were excluded.

### Search Results

**FIGURE 1:**
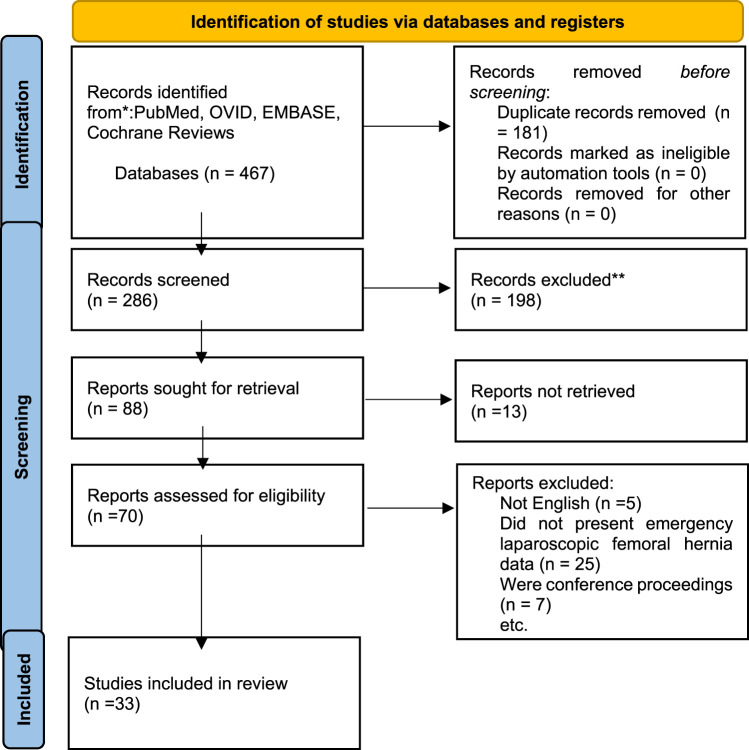
Prisma Diagram, from (43).

Results returned from each search are shown in the PRISMA diagram in Figure 1.

OVID Medline 94, OVID Embase 206, Pubmed 167, Duplicates 181, Cochrane database 0.

286 unique papers were identified. Papers were screened by title and abstract prior to inclusion or exclusion. After abstract screening there were 87 articles potentially eligible. These articles were reviewed in full to assess for eligibility for inclusion. After review of the papers 5 further papers were identified from the reference sections.

24 case reports, and 9 papers were identified.

Case reports were summarised and tabulated extracting data for the characteristics as shown in Table 1. Case series and cohorts studies were summarised in Table 2.

**TABLE 1:** Summary of case reports.

Author	Year	Country	Sex	Age	Side	Hernia contents	Method of hernia repair/material	Mesh(type, size, fixation)	Resected organ	Follow up	Discharge day	MIS repair	Role of laparoscopy
Luo et al. (10)	2022	China	F	65	R	Proximal small bowel	TAPP, mesh	Polypropylene	none	none	soon	yes	Hernia repair, diagnostic
Alkashty et al. (11)	2021	England	M	80	R	Appendix	Laparoscopic sutured vicryl, then open plug	plug (unspecified)	appendix	3 months	1	hybrid	Appendicectomy, hernia repair
Tsuchiya et al. (12)	2021	Japan	M	81	L	Small bowel	Laparoscopic sutured	None	small bowel	1 year	8	yes	Lap sutured repair, diagnosed small bowel ischaemia
Sartori et al. (13)	2020	Italy	F	63	R	Appendix	TAPP, mesh	16 cm × 12 cm Progrip	appendix	3 months	3	yes	Appendicectomy, hernia repair
Sharma et al. (14)	2020	USA	F	64	R	Appendix	Open, sutured (McVay)	None	appendix	none	2	no	Division of appendix base, appendicectomy through the groin
Simpson et al. (15)	2020	USA	F	84	R	Appendix	Open, mesh	plug, 6 layer Acell Gentrix	appendix	none	2	no	Hernioscopy, lap appendix
Chouari et al. (16)	2020	England	F	84	R	Appendix, omentum	Open, sutured repair	None	appendix, omentum	none	3	no	Appendicectomy
Lee et al. (17)	2019	England	F	72	R	Appendix	Open, sutured	None	appendix	10 weeks	1	no	Appendicectomy
Namba et al. (18)	2019	Japan	f	75	R	Small bowel	TEP, mesh	10x15 TiLENE	none	none	not stated	yes	Diagnostic of small bowel viability, TEP hernia repair
Rollo et al. (19)	2019	Italy	F	82	R	Appendix	Laparoscopic sutured	Sutured, unspecified	appendix	none	8	yes	Diagnostic, appendicectomy, hernia repair
Kafadar et al. (20)	2018	Turkey	F	45	R	Distal Jejenum	TAPP, mesh	Prolene, size unspecified	none	12 months	3	yes	Reduction of small bowel, inspection, TAPP repair
Ikram et al. (21)	2018	England	F	71	R	Appendix	TAPP, mesh	“Small, composite”	appendix	none	2	yes	Appendicectomy, hernia repair
Sinclair et al. (22)	2018	England	F	81	R	Appendix and small bowel	Laparoscopic sutured	None	appendix	none	9	no	Appendicectomy, small bowel inspection, repair
Kim et al. (23)	2017	Singapore	F	63	L	Small bowel	TEP, mesh	10 cm × 15 cm TiLENE	none	6 months	not stated	yes	TEP repair, converted to laparoscopic SB inspection
Klipfel et al. (24)	2017	France	F	77	R	Appendix	TAPP, mesh	Tutomesh (biologic)	appendix	1 month	2	yes	Appendicectomy, hernia repair
Soeta et al. (25)	2017	Japan	F	85	R	Small bowel, omentum	Open, Kugel patch	Unspecified	small bowel	15 months	10	no	Diagnostic, reduction of hernia
Sibona et al. (26)	2016	USA	F	35	R	Appendix	Open, sutured repair, vicryl	None	appendix	none	2	no	Diagnostic, appendicectomy
Pillay (27)	2015	Canada	F	45	R	Omentum	TAPP, mesh	Unspecified	none	none	2	yes	Diagnostic, hernia repair
AlSubaie et al. (28)	2015	Kuwait	F	59	R	Appendix	TAPP, mesh	15 cm × 15 cm Prolene	appendix	4 weeks	2	yes	Diagnostic, appendicectomy, hernia repair
Valderrama et al. (29)	2014	USA	F	64	R	Small bowel	Open, plug mesh	Plug, unspecified	none	short	not stated	no	Diagnostic, bowel assessment
Ginesta et al. (30)	2013	Spain	F	94	L	Small bowel	TEP, mesh	Polypropylene, unspecified	small bowel	6 months	4	yes	Diagnostic, TA reduction of hernia and SBR, TEP repair
Thomas et al. (31)	2009	USA	F	77	R	Appendix	Open, sutured, polypropelene	None	appendix	short	2	yes	Appendicectomy
Comman et al. (32)	2008	Germany	F	64	R	Meckle’s	TAPP, plug mesh	Plug, unspecified	Meckle’s	unspecified	5	yes	Diagnostic, Meckle’s resection, repair
Comman et al. (33)	2007	Germany	F	38	R	Appendix	TAPP, mesh	10x15 polypropylene	appendix	14 days	1	yes	Diagnostic, appendicectomy, hernia repair

**TABLE 2:** Summary of case series and cohort studies.

Author	Type of paper	Subjects	Technique	Outcomes	Limitations	Findings
Lin 2001 (34)	Case series	2	“hernia sac laparoscopy”	Descriptive only	Limited data, not repairing the hernia laparoscopically	Presents the role of diagnostic hernioscopy
			Open repair			
Yau 2007 (35)	Case series	8, all lap femoral hernias	TAPP with a mesh plug	Length of stay, recurrence, complications	Small numbers	No recurrences (8–18 months follow up, median 13 months)
					No randomisation	Median LOS 2 days
						No complications or conversions
Sasaki 2014 (36)	Case series	4 cases, 2 femoral	TEP	Descriptive only	Small numbers	No significant complications
			Delayed hernia repair after laparoscopic small bowel resection		No indication of patient selection	
					No follow up data	
Leung 2012 (37)	Case series	47 cases, of which 10 were femoral	37 TEP	Length of stay	Outcomes not differentiated between inguinal and femoral hernias	Mean follow up 14 months with no recurrence
			4 TAPP	Recurrence		Mean LOS 1.7 days
			2 other			
Clyde 2018 (38)	Cohort study	Unclear, mixed data, likely 6 lap femoral	Mixture of TEP, Low and high open approaches	Recurrence within 5 years	Mixed elective and emergency data	No difference in recurrence between mesh or sutured repairs
					No reason given for open or MIS choice	
Rebuffat 2005 (39)	Cohort study	1532, of which 40 femoral, 7 laparoscopic emergency cases	TAPP	Length of stay, recurrence rate, complications	Data for inguinal and femoral hernia presented together, low number of femoral hernias	Limited by the heterogeneity of the data
Chihara 2018 (40)	Cohort study	106 total, 30 femoral, 17 open 13 laparoscopic	106 cases, 30 femoral, 13 laparoscopic	Complications	17 years collection of data with a change mid study	Significantly lower complications in MIS group (18.%/3.9% *p =* 0.172)
				Length of stay	Heterogenous data	LOS 5.6/14.7 days lower in MIS group
Andreson 2005 (8)	Cohort study	3970 primary femoral hernia repairs	Laparoscopic, not discriminated as TAPP/TEP	Re-operation rate	Limited emergency MIS	Laparoscopic protective against further inguinal hernias
		1557 as an emergency			No randomisation or reasoning for operation choice	Female sex, open repair independent risks for re-operation
		57 laparoscopic				
Dahlstrand 2009 (41)	Cohort study	3980 femoral hernia repairs	Laparoscopic, not discriminated as TAPP/TEP	30 days mortality	MIS repair techniques unclear	No significant difference in outcomes between different techniques in the emergency cohort
		1430 emergency		Reoperation within 5 years	No randomisation	No difference in reoperation rate with or without mesh
		24 laparoscopic			No reasoning for operative choice	

## Results

### Case Reports

24 case reports were identified as outlined in Table 1. Data has been included when stated in the case report. Reports are more common recently but they range from 2008 to 2022. There were older case reports than this, but we were unable to retrieve the full papers. The most common country reporting cases was the UK with 5 (21%) of reports. There was a female to male ratio of 22:2, and hernias were much more likely to be right sided at 21:3. Median age was 71.5 (range 35–94).

The most common procedure was a laparoscopic appendicectomy due to De Garengeot’s hernia with a mixture of hernia repairs *via* an open, or minimal access approach. There were no cases managed robotically reported. There will be an element of reporting bias as this would be a relatively unusual finding and more likely to be reported.

None of the case reports indicate why they chose a laparoscopic approach in particular and there was no randomisation.

There were no significant complications and the length of stay was usually short, with most patients being discharged on day 1–2. The longer lengths of stay were associated with hernias containing small bowel. None of the cases reported any conversions to open surgery.

### Case Series

#### 4 Short case series were identified as eligible for inclusion

Lin et al (2001) presented data for 5 patients operated on as an emergency due to incarcerated groin hernias with small bowel obstruction. 3 patients were inguinal, 2 femoral, a 79 years old female and a 82 years old female. Both were right sided. They were managed *via* open hernia repair with plug repairs, and “hernia sac laparoscopy.” They inserted a 10 mm 0° laparoscope *via* the hernia sac to examine the small bowel reduced from the hernia in order to avoid laparotomy. Both patients were discharged within 3 days. There were no reported complications (34).

Yau et al. (2007) presented 8 consecutive patients operated between July 2003 and November 2005 for incarcerated femoral hernias. Patients were excluded if there was evidence of peritonitis, or if they had previously had more than one abdominal operation; these patients were operated on by open approach. There were 7 female and 1 male patients with 5 hernias left sided and 3 right sided. 5 hernias were found to contain omentum, 3 small bowel; no resections were required. A standard transabdominal laparoscopic approach was used, with placement of a prolene mesh plug filling the defect. Median length of stay was 2 days (range 1–4). Median follow up was 13 months (range 8–18) with no recurrence observed, although how this was assessed was not described. There were no significant complications (33).

Sasaki et al. (2014) reported 4 patients with strangulated hernias managed by TEP. They presented a 2-stage hybrid approach with laparoscopic release of the hernia and bowel resection, followed by TEP repair of the hernial defect between 8 and 24 days later. 2 of the 4 patients had femoral hernias. The first case was an 86 years old female with left sided femoral hernia, who had been managed non-operatively at another site for 10 days prior to transfer. 24 days post laparoscopic small bowel resection a TEP repair was performed using a 7.7 cm × 12.6 cm polypropylene mesh (Surgipro, Covidien). She was discharged on day 10. The second case was a 82 years old female managed with TEP 13 days post laparoscopic small bowel resection. She was discharged 5 days after the second surgery. There were no significant complications in either of these patients, or the other two patients who had obturator and inguinal hernias. The rationale of the authors was to avoid mesh infection, and allow the usage of synthetic mesh, by separating the small bowel resections and hernia repairs both anatomically in different planes, and at different episodes. No follow up data was presented (36).

Leung et al. (2012) reported 47 cases of strangulated groin hernias managed laparoscopically as an emergency between Jan 2007 and Dec 2009 with a mixture of TEP (37) and TAPP (4) repairs with 2 “Board ligament” (sic) repairs. Exclusion criteria included scrotal hernias, extensive previous surgery, medical comorbidities precluding general anaesthetic. 10 hernias were femoral, 36 inguinal, 3 obturator. 32 hernias contained small bowel, 2 patients required a resection, 1 due to perforation reducing the hernia, and 1 due to ischaemia secondary to an obturator hernia. There were no major complications reported, although the one patient with a prolonged stay secondary to a chest infection had a femoral hernia. Mean length of stay was 1.7 days (1–5) for under 60s and 3.5 for over 60s (1–17). There were no conversions to open surgery. Mean follow up was 14 months with no recurrences. Outcomes are not differentiated by hernia type and therefore it is difficult to draw any significant conclusions pertaining specifically to femoral hernias. Recurrence was not defined and there was no randomisation with it being unclear if there were other patients operated on *via* an open approach (37).

### Cohort Studies

Clyde et al. (2018) reported a retrospective review of 297 consecutive cases prospectively collected, of primary femoral hernia repairs, between 2007 and 2013. Patients who were uncontactable were excluded, leaving 138 patients included in the study. Their primary outcome was recurrence, particularly looking at the role mesh played. This was defined as an ipsilateral groin swelling confirmed at outpatient follow up, or on patient reported symptoms during a telephone interview as part of the audit. Telephoned patients were then reviewed in person, and recurrence confirmed clinically in 80% of cases. Repairs were categorised as low (Lockwood) approach, high (McEvedy approach) or TEP, no TAPP repairs were performed. TEP repairs were performed without mesh fixation. They presented data for both elective and emergency cases. Within the 138 patients included, 45 were operated on as an emergency, and 47 by TEP. It is not possible to discern what proportion of the TEP cases were operated on electively as the emergency and electively data was not separated by approach. Mesh was used in only 6 emergency cases, but was used in all 47 TEP repairs, implying that there were few emergency TEP repairs, and the vast majority were elective. Their primary outcome of recurrence showed no significant difference in recurrence rates between the various operation techniques, use of mesh or primary sutured repair, or between patients operated on electively or as emergency. There was no indication as to why an open or laparoscopic approach was chosen, and no randomisation. The follow up was relatively long as few studies in femoral hernias have a 5 years follow up (38).

Rebuffat et al. (2005) presented prospective data on strangulated groin hernias having reviewed a prospectively collected database of 1532 consecutive TAPP hernia repairs. There were 28 emergency cases, of which 7 were femoral hernias. There were no major “complications” with one patient having a haematoma. Mean length of stay was 3.9 days (0–38) and small bowel resection was required in 7 cases. Mean follow up was 340 days with no recurrences noted in the follow up period. There were 3 conversions (10.7%) 1 because of extensive adhesions, 2 because of a lack of space due to intestinal distension, it is unclear if these were femoral or inguinal hernias. Data for the outcomes of the emergency repairs is not presented separately for femoral and inguinal hernias so again, no significant conclusions can be drawn relevant to femoral hernias particularly other than they did not appear to have any major differences in outcomes compared with the inguinal hernias (39).

Chihara et al. (2018) reported prospectively collected data for 106 patients with incarcerated groin hernias, 30 femoral, of which 17 had open operations, and 13 laparoscopic. The study period was between 2000 and 2017, adopting a laparoscopic approach for the second half of the study period, and the two arms did not run concurrently. The two groups were comparable with no statistically significant difference in age, sex, BMI, ASA or hernia type. They compared open and laparoscopic cases and presented data without specifying the hernia type. Whilst their data does not pertain only to femoral hernias there were some interesting conclusions. They showed a significant reduction in post operative complications in the laparoscopic cohort (18.%/3.9% *p =* 0.172), with 2 bladder injuries in the open group. They do not report the experience or grade of the surgeons associated with these particular cases. There was 1 conversion in the laparoscopic (TAPP) group due to a large inguinoscrotal hernia being unmanageable laparoscopically. They also recommended a 2 stage approach in the presence of perforation or pus, with laparoscopic sutured hernia repair, and a delayed mesh repair at a later date. This was performed in 7 patients with no significant complications. The length of stay in the laparoscopic group was shorter (5.6 vs. 14.7 days). There will have been many changes in medical practice during the 17 years of the study and there any many potential sources of bias around their management (40).

Most of the data presented in these studies is not presented separately either for femoral and inguinal hernias, or elective and emergency case, and therefore unfortunately only limited conclusions regarding femoral hernias specifically can be made, and a meta-analysis cannot be performed. They appear to show no major differences in outcomes between femoral and inguinal hernias, but the numbers are low and would be underpowered unless differences in outcomes were large.

### National Hernia Registry Studies

#### Danish Hernia Database

Andreson et al. (2005) (8) presented a cohort study comparing outcomes of open vs. laparoscopic repair of femoral hernias from the Danish Hernia Database between Jan 1998 to Feb 2012 comprising of 3970 total primary cases. The main outcome measure was re-operation assessed by analysing based on each patient’s unique social security number meaning recurrences were identified even this was operated on at a different hospital. A total of 1557 (39.22%) emergency procedures took place during the study period of which 57 (3.66%) were laparoscopic. 2/57 (3.5%) patients from the emergency MIS cohort required re-operation, one for recurrent femoral hernia, one for ipsilateral inguinal hernia, compared with 10/454 (2.2%) from the elective MIS and 66/1500 (4.4%) from the open emergency group. They found no significant difference between the re-operation rate between elective and emergency cases generally. Inguinal hernias were statistically significantly more likely to be found at re-operation in open cases than laparoscopic cases (*p* < 0.001) but this was not stratified for emergency or elective. The main findings were that an open repair, and female sex, were independent risk factors for re-operation after femoral hernia repair, and that laparoscopic operations are protective for requiring an operation for an inguinal hernia at a later date. Their data also showed a significant shift from an open repair being standard in 1998 with less than 5% of cases being laparoscopic, to 70.3% of cases being performed laparoscopically in 2011. It is expected this trend has continued (8). Because of the nature of registry reviews there was no randomisation, or explanation regarding the choice of operative technique.

#### The Swedish Hernia Register

Dahlstrand et al. (2009) (41) analysed the register for cases between 1992 and 2006 presenting data of 3980 femoral hernia repairs, 1430 (35.92%) performed as an emergency, of which 24 had a laparoscopic pre peritoneal mesh repair. From the data presented it’s not clear if there were any laparoscopic sutured repairs. Data was analysed for 30-day mortality, and re-operation within 5 years. The table below shows the re-operation rates of the various approaches. In the elective cohort mesh repairs were statistically less likely to require re-operation for recurrence than sutured repairs. No approach showed any statistically significant advantage over others in the emergency cohort, and the use of mesh did not show any superiority over sutured repairs, although the emergency laparoscopic group has particularly low numbers. The 30-day Standardised Mortality Rate after an emergency repair was 7 times higher than the baseline Swedish population. Bowel resection was also associated with increased mortality risk. Again, the laparoscopic numbers are too low to make any firm conclusions (see Table 3).

**TABLE 3:** Showing reoperation risk, by surgical approach

Type of repair	Reoperated n/No at risk (%)	Univariate model HR (95%CI)	Reoperated n/No at risk (%)	Univariate model HR (95%CI)
	Elective		Emergency	
Open Suture	60/938 (6.4)	1 (ref)	44/930 (4.7)	1 (ref)
Mesh plug	18/436 (4.1)	0.73 (0.43–1.25)	5/176 (2.8)	0.68 (0.27–1.71)
Inguinal mesh	23/553 (4.2)	0.88 (0.51–1.43)	11/173 (6.4)	1.57 (0.81–30.4)
Preperitoneal mesh (open)	6/250 (2.4)	0.47 (0.2–10.8)	8/106 (7.5)	1.74 (0.82–3.70)
Preperitoneal mesh (lap)	8/347 (2.3)	0.45 (0.21–0.94)	1/24 (4.2)	0.86 (0.12–6.25)

Adapted from (41).

Both papers assessing the large national databases are relatively old. Minimal access surgery has progressed significantly over the last 20 years and their findings may not be applicable anymore.

## Discussion

The authors have chosen to include all the published data on MIS in emergency femoral hernia repairs identified by the search strategy. There were no papers identified specifically reporting on the management of femoral hernias as an emergency by an MIS technique other than the small case series by Yau (35). The data is generally very heterogenous, often presenting a mixture of inguinal and femoral hernias together, or not discriminating between elective and emergency cases. As such no meta-analysis or statistical tests can be performed. The 2 large national database studies show the very low number of femoral hernias managed laparoscopically as an emergency in Denmark and Sweden during the examined period. MIS techniques are likely to be employed more frequently now as demonstrated by the trends in the Danish Hernia Registry reported by Andreson (8). Prospectively collected databases such as the European Hernia Society registry, and the significant change towards femoral hernias being managed laparoscopic routinely electively, should see a major change should these registers be re-examined for emergency cases.

There are no randomised controls trials relating to the topic, and the data gleaned is generally of low quality.

### The Role of Diagnostic/Therapeutic Laparoscopy

Within the 24 case reports there was significant variation in the role laparoscopy played. In 15 of the cases the repair was performed *via* a laparoscopic technique, 9 TAPP, 3 TEP, 3 sutured with 1 sutured and then an open plug repair. There was also a significant role played in the management of the hernia sac contents, primarily in the treatment of appendicitis, but also small bowel resection. The important diagnostic role of an MIS approach, allowing sac contents to be examined without the need for lower midline laparotomy should be appreciated. 6 out of 24 cases included resection of sac contents laparoscopically. Presumably this would have been more difficult in most of these cases through a groin incision. There will be an element of selection bias simply because a case is more likely to be reported if it is interesting and unusual, and therefore simple hernia repairs are less like to be reported.

Within the reported cohort studies and case series there is a low conversion rate, with few intra operative complications. There was no statistically significant difference between the conversion rates of elective and emergency cohorts in any of the papers suggesting that a laparoscopic approach is feasible and technically achievable.

There is little evidence to support TEP or TAPP over one another. Several of the case reports discussed the benefit of TEP for obstructed small bowel hernias, where they found that there was more space in the extraperitoneal plane. In these cases, they examined the hernial contents *via* traditional laparoscopy, and converted to TEP for the mesh placement. Both conversions in Rebuffat’s (39) case series were because of a lack of space, attempting TAPP repair of an obstructed small bowel hernia. Logically this approach should reduce mesh infection by separating the mesh from potentially infected sites, but there is no evidence to support this. In the author’s opinion it is harder to assess the viability of hernia contents adequately *via* a TEP approach. The larger registry studies do not report on conversion rates. The author’s can tentatively suggest that in the presence of small bowel obstruction and dilated bowel that a TEP repair may be more easily achievable. Logic would suggest a reduced rate of small bowel injury if the peritoneal cavity is not entered, but there is no evidence to support this statement.

### Recurrence and the Use of Mesh

Dahlstrand (41) was unable to demonstrate a significant advantage for any technique in the emergency setting implying that laparoscopic repair is at least non-inferior to open sutured repair in terms of recurrence alone, although numbers were low and may well be underpowered except for large differences. The number of laparoscopic cases in the emergency cohorts in all the papers were low, a re-examination of the Swedish and Danish Hernia registers, specifically looking at the emergency cases, would likely find many more cases now with a further 10–15 years of practice.

There was no significant evidence presented regarding long term chronic pain or quality of life assessment. Within the setting of emergency femoral hernia repairs this raises the question of the requirement for mesh, given there is no demonstrable reduction in recurrence rate, and the current political climate around mesh and mesh complications. There was no evidence of an increased mesh infection rate in any of the papers or case reports when compared with elective cases to suggest it should be avoided in the emergency setting. Mesh has previously been shown to be safe in the emergency management of inguinal hernias (42) and logically this could be extrapolated to femoral hernias if a benefit of mesh use was demonstrated.

### Length of Stay

Within the case reports the median length of stay was not stated in 4/24 cases, and only stated as “soon” in another. The median length of stay in the others was 2 (range 1–10) None of the case series differentiated data adequately to find the length of stay for emergency laparoscopic femoral hernia repairs. It appears that the length of stay in patients operated on laparoscopically is at least as good as that *via* an open approach. The case series and cohort studies presented the data without discriminating adequately as to allow comment on the length of stay in emergency MIS cases in particular, although where stated length of stay was lower in the MIS cohorts. The benefits of MIS have been established elsewhere in regards to length of stay and return to normal function.

### Complications

There were no increased complication rates associated with laparoscopic surgery in any of the large series. Chihara (40) found a statistically significantly lower complication rate in the laparoscopic cohort, but this was a small series and could be skewed by the two complications (bladder injury) in the open cohort. There is no evidence to suggest that laparoscopic management is unsafe. There are no comments in any of the papers regarding surgical site infections or long-term complications such as adhesions or port site hernias.

### Quality of Life

None of the eligible papers or case reports included any assessment of quality of life, or return to normal function, but they mostly included a length of stay which was usually quite short. There is no new data presented in the case series or the Hernia registry papers. However, there are no major complications or problems on follow up, suggesting that the benefits of MIS should still pertain to an emergency cohort as established already in the elective setting, combined with an avoidance of laparotomy where assessment of the hernia contents is required.

## Limitations

The authors have decided to include the case reports as a complete summary of what evidence there is published on the topic. They are included to demonstrate the role of laparoscopy in managing femoral hernias *via* a hybrid approach, and to raise awareness of the potential role of minimal access surgery to contribute diagnostically, whilst managing the hernia in a conventional manner.

There are no randomised control trials published and no studies published where the emergency management of femoral hernias is presented as a primary objective of the trial. Data from the cohort studies and the large national databases do not present femoral hernias separately, and often only as a very minor aspect of what they are presenting. Therefore, it is impossible to extract any conclusive data specifically regarding the emergency management of femoral hernias *via* a laparoscopic technique. Data presented is heterogenous and frequently non-specific, meaning no meta-analysis, or statistical tests can be performed.

A randomised control trial, or re-examination of the large hernia registries, specifically looking at the emergency management of femoral hernia by MIS techniques is required before any firm conclusions can be made.

## Conclusion/Recommendations

A minimal access approach can safely avoid the need for laparotomy aiding the identification and management of strangulated hernias through a thorough inspection of all intra-peritoneal organs, especially *via* TAPP approach, or a hybrid approach *via* hernioscopy or diagnostic laparoscopy. Femoral hernias are being managed *via* a laparoscopic approach by many centres, but there is very limited data published specifically on this topic and outcomes are unknown.

There is currently no evidence to support the use of mesh in the management of femoral hernias as an emergency, in terms of reducing recurrence rates. More studies ought to be conducted, particularly using data from national registries which could be extracted looking specifically at this. Data from the large national databases currently suggests a similar recurrence rate with and without mesh when femoral hernias are managed emergently.

The evidence in favour of a minimally invasive surgical approach in emergency femoral hernias is lacking to date, therefore more studies where the outcome in this category of patients is compared with classical open approach is needed.

On the evidence to date, we can conclude that a laparoscopic approach in emergency femoral hernia repair appears safe and technically feasible, but there is little evidence to support an MIS approach over established open techniques, with very little published on the subject. What is published appears to suggest it is a safe and viable option.
